# Editorial: Doubled Haploidy in Model and Recalcitrant Species

**DOI:** 10.3389/fpls.2015.01175

**Published:** 2015-12-24

**Authors:** Jose M. Seguí-Simarro

**Affiliations:** Cell Biology Group, COMAV Institute, Universitat Politècnica de ValénciaValencia, Spain

**Keywords:** plant breeding, haploidy, doubled haploidy, gynogenesis, androgenesis

Doubled haploid (DH) technology is a powerful tool in plant breeding to reduce the time and costs needed to produce pure lines, the cornerstone of hybrid seed production. This biotechnological alternative to classic methods allows for a reduction of the typical 7–8 inbreeding generations needed to fix a hybrid genotype to only one *in vitro* generation. It is therefore much faster and cheaper, being the principal advantage of DH technology in plant breeding, but not the only. Indeed, DHs are also useful for genetic mapping of complex qualitative traits, for linkage studies and estimation of recombination fractions, to unmask recessive mutants, to avoid transgenic hemizygotes, or for reverse breeding, among others (Forster et al., 2007; Dunwell, 2010; Dwivedi et al., 2015). These are some of the advantages that make DH technology one of the most exciting fields of present and future plant biotechnology.

At present, there are several ways to produce haploids and eventually DHs (after a process of chromosome doubling), involving both female and male gametophytes. From the female gametophyte, haploids may be produced by uniparental genome elimination and by induction of gynogenesis. Uniparental genome elimination is typically achieved by crossing two sexually incompatible species, in some intraspecific crosses when one genitor carries specific mutation(s), or through genetic manipulation of CENH3, a centromeric variant of the H3 histone (Ravi and Chan, 2010, 2013; Karimi-Ashtiyani et al., 2015). Gynogenesis is a route through which unfertilized ovules, ovaries or even entire flowers are cultured *in vitro* to induce the development of a haploid embryo, generally from the egg cell (Bohanec, 2009). From the male gametophyte, haploids may be obtained through androgenesis (Seguí-Simarro, 2010). The most common and useful androgenic pathway is microspore/pollen embryogenesis, through which microspores/pollen are reprogrammed toward embryogenesis. Discovered more than 40 years ago (Guha and Maheshwari, 1964), this process has become of great practical importance for the agronomic industry due to its convenience for producing DH lines much faster, cheaper, and in more species than the other methods above mentioned (Forster et al., 2007; Dunwell, 2010). This is why when possible, microspore embryogenesis is the method of choice to produce DHs.

For all these methods, there are species where they are most efficient. This is why these species are used as experimental models to study basic aspects of the process. This is the case of onion for gynogenesis, and of barley and rapeseed for microspore embryogenesis in monocots and dicots, respectively. This Research Topic includes examples of research focused on different aspects of gynogenesis and microspore embryogenesis in these species. For example, Fayos et al. compare the performance of different onion germplasms under different experimental conditions to induce gynogenesis, regenerate gynogenic plants, and promote chromosome doubling. As to microspore embryogenesis, several articles use barley to study it. Daghma et al. develop a time-lapse imaging system to track the first embryogenic divisions, finding that most embryogenic structures come from symmetrically divided vacuolated microspores, with very few coming from asymmetric divisions. Lippmann et al. develop a micro-culture system whereby they demonstrate that co-cultivated wheat pistils release a low molecular weight signal that increases considerably the production of embryogenic structures. They postulate that the use of cut pistils as sources of this feeder substance might be extended to other species. Solís et al. use 5-azacytidine, a non-methylable base analog, to study the effects of DNA hypomethylation during microspore embryogenesis. They find that hypomethylation promotes the developmental switch toward proliferation, but prevents further differentiation into true embryos, both in barley and rapeseed. Also in rapeseed, the use of the most advanced sample preservation techniques allowed for the discovery of new processes associated to the embryogenic switch. In Parra-Vega et al., and Parra-Vega et al., the authors report the occurrence of plastolysomes (autophagic plastids) that engulf and digest cytoplasm regions, being finally released to the apoplast. They also describe the parallel formation of a callosic layer beneath the microspore intine, and the *de novo* formation of abnormal cell walls with altered callose and cellulose composition. All these events appear to have a dramatic impact in the developmental fate of the embryogenic microspore, including genome duplication by nuclear fusion.

However, not all the species respond well enough to DH technology. Indeed, many of them are still considered as recalcitrant to these treatments, including many of the most important crops worldwide. Despite the work of many groups, little is still known about how to overcome recalcitrancy. This is why it is also important to shed light on the particularities of recalcitrant species and the special conditions they need to be induced. In this Research Topic, Castillo et al. show that preconditioning or coculture with ovaries increases the efficiency of DH production and chromosome doubling in different bread wheat cultivars, being the increases higher in those most recalcitrant. Interestingly, these results are in line with those from Lippmann et al. about the role of the female parts in helping the development of microspore-derived embryos in an *in vitro* environment, devoid of the complex crosstalk between embryo, endosperm and seed tissues that takes place during zygotic embryogenesis. In this crosstalk, hormones play a key role. This is why Żur et al. present a review focused on the current knowledge of hormonal regulation during microspore embryogenesis. Besides the role described for the principal hormones, either when they act endogenously or when applied exogenously, this review presents new and interesting notions about their involvement in this process. A remarkable example of the application of this kind of knowledge is the study brought by Chiancone et al., which achieves an important milestone inducing for the first time the development of microspore-derived embryos in different cultivars of *Citrus*, a very recalcitrant fruit crop, through the use of meta-topolin, a plant hormone rarely use in microspore embryogenesis.

Together, the papers of this Research Topic show some relevant advances in the understanding of the processes that lead to the formation of DH plants, and in their application to improve its performance in recalcitrant genotypes.

## Funding

This work was supported by grant AGL2014-55177-R from Spanish MINECO to JMSS.

### Conflict of interest statement

The author declares that the research was conducted in the absence of any commercial or financial relationships that could be construed as a potential conflict of interest.

## References

[B1] Bohanec B. (2009). Doubled haploids via gynogenesis, in Advances in Haploid Production in Higher Plants, ed Touraev A. F. (New York, NY: Bp Jain; Sm; Springer), 35–46.

[B2] Dunwell J.M. (2010). Haploids in flowering plants: origins and exploitation. Plant Biotechnol. J. 8, 377–424. 10.1111/j.1467-7652.2009.00498.x 20233334

[B3] Dwivedi S. L. Britt A. B. Tripathi L. Sharma S. Upadhyaya H. D. Ortiz R. (2015). Haploids: Constraints and opportunities in plant breeding. Biotechnol. Adv. 33, 812–829. 10.1016/j.biotechadv.2015.07.001 26165969

[B4] Forster B. P. Heberle-Bors E. Kasha K. J. Touraev A. (2007). The resurgence of haploids in higher plants. Trends Plant Sci. 12, 368–375. 17629539 10.1016/j.tplants.2007.06.007

[B5] Guha S. Maheshwari S. C. (1964). *In vitro* production of embryos from anthers of *Datura*. Nature 204, 497.

[B6] Karimi-Ashtiyani R. Ishii T. Niessen M. Stein N. Heckmann S. Gurushidze M.. (2015). Point mutation impairs centromeric CENH3 loading and induces haploid plants. Proc. Natl Acad. Sci. U.S.A. 112, 11211–11216. 10.1073/pnas.1504333112 26294252 PMC4568683

[B7] Ravi M. Chan S. W. L. (2010). Haploid plants produced by centromere-mediated genome elimination. Nature 464, 615–618. 10.1038/nature08842 20336146

[B8] Ravi M. Chan S. W. L. (2013). Centromere-mediated generation of haploid plants, in Plant Centromere Biology, eds Jiang J. Birchler J. A. (Hoboken, NJ: John Wiley & Sons), 169–181.

[B9] Seguí-Simarro J. M. (2010). Androgenesis revisited. Bot. Rev. 76, 377–404. 10.1007/s12229-010-9056-6

